# Liquid PTVA: a faster and cheaper alternative for generating multi-copy clones in *Pichia pastoris*

**DOI:** 10.1186/s12934-016-0432-8

**Published:** 2016-02-05

**Authors:** Rochelle Aw, Karen M. Polizzi

**Affiliations:** Department of Life Sciences, Imperial College London, London, SW7 2AZ UK; Centre for Synthetic Biology and Innovation, Imperial College London, London, SW7 2AZ UK

**Keywords:** *Pichia pastoris/Komagataella phaffii*, Multi-copy clones, Posttransformational vector amplification, Recombinant protein expression

## Abstract

**Background:**

Multiple cognate gene copy clones have often been used in order to increase the yield of recombinant protein expression in the yeast *Pichia pastoris*. The method of posttransformational vector amplification (PTVA) has allowed for the efficient generation of multi-copy clones in *P. pastoris*. However, despite its relative ease and success, this process can be expensive and time consuming.

**Results:**

We have developed a modified version of PTVA, called Liquid PTVA, which allows for faster and cheaper selection of multi-copy clones. Cultures are grown in liquid medium with only a final selection carried out on agar plates, reducing overall antibiotic usage and increasing the speed of clone amplification. In addition, it was established that starting PTVA with a single copy clone resulted in higher copy number strains for both traditional plate PTVA and liquid PTVA. Furthermore, using the Zeocin selection marker in liquid PTVA results in strains with higher growth rates, which could be beneficial for recombinant protein production processes.

**Conclusions:**

We present a methodology for creating multi-copy clones that can be achieved over 12 days instead of the traditional 45 and at approximately half the cost.

**Electronic supplementary material:**

The online version of this article (doi:10.1186/s12934-016-0432-8) contains supplementary material, which is available to authorized users.

## Background

*Pichia pastoris* has been used for over 30 years to produce recombinant proteins with expression levels of select proteins reaching up to 20 g L^−1^ [1, 2]. *P. pastoris* is an ideal industrial cell factory due to its ability to reach very high cell densities and secrete proteins into the supernatant, which coupled with a low level of native proteins, eases downstream processing [3]. A popular way to create strains with increased protein production is to increase the number of cognate genes [4, 5]. This is particularly effective with intracellular expression of proteins [6, 7], but is also a common strategy with secreted proteins [8, 9].

While an increase in titer from multi-copy clones has often been reported, there are instances, especially with secreted proteins, where the relationship is not linear (i.e. the highest copy strains do not always give the highest expression) [5, 10]. This is often attributed to bottlenecks in the secretory pathway [11]. However, the copy number at which secretion saturation occurs is often protein-specific, and as a result strains with different copy numbers must be evaluated in order to identify those with the maximum expression [7]. Furthermore, increasingly strain engineering efforts have aimed to expand the capacity of the secretory pathway, e.g. by overexpressing accessory proteins to aid in protein folding [12, 13]. Such research relies on testing expression at a range of copy numbers to show the effect of the engineering efforts on the titer obtained. Thus, there is a need for a rapid and reliable method to generate strains with a range of gene copy number.

There are several established experimental methods for generating multi-copy clones including in vitro multimerization of the vector before transformation and direct selection of transformants on high concentrations of antibiotics, made possible by the increased use of Zeocin and the modification of the *Tn903kanr* gene meaning pre-selection using histidine auxotrophy is not required [14]. With the direct selection method, the number of colonies generated on plates containing higher concentration of antibiotics is often severely reduced, limiting the number of multi-copy strains obtained. However, a higher proportion of the surviving population will be multi-copy clones and so such experiments can still be used to generate strains with a range of copy number.

Due to the low efficiency of generating multi-copy clones via direct selection, in 2008 Sunga et al. proposed the method of posttransformational vector amplification (PTVA). In PTVA, instead of direct selection on high concentrations of antibiotic, cells are spotted onto agar plates with increasing antibiotic concentrations with approximately 5 days growth in between each step [15]. During the outgrowth phase, the copy number of the antibiotic resistance gene is increased to allow the cell to adapt to the higher antibiotic concentration. Using Southern blot, it was demonstrated that cells actually amplify the entire cassette, including the gene of interest. Thus, strains that survive at higher antibiotic concentrations also contain a higher number of intact copies of the gene of interest. The advantage of using PTVA over direct selection is that the frequency of “jackpot” clones, those with over 10 copies, increases from 1–2 to 5–6 % [15].

PTVA has been widely adopted by the *P. pastoris* community with many studies using it for comparison of titers from strains of different copy number [7, 16, 17]. However, despite the apparent ease of PTVA, the methodology can be time consuming and laborious, not to mention expensive, particularly when Zeocin is used as a selection agent. Herein, we describe a method to reduce the time and cost to carry out PTVA through serial passaging in liquid medium, which still results in a wide range of strains containing different copy numbers.

## Results and Discussion

### Liquid PTVA results in multi-copy clones with saturated GFP expression

#### Liquid PTVA with a medium change every 12  h versus Plate PTVA

Initially four individual vectors were designed: pZGFP, pZαGFP, pKGFP and pKαGFP, all expressing the green fluorescent protein (GFP) under the control of the alcohol oxidase 1 (AOX1) promoter (Additional file 1: Figure S1). pZGFP and pZαGFP utilize the commercial pPICZ and pPICZα vectors from Invitrogen, respectively, while pKGFP and pKαGFP use the pKANB and pKANαB vectors, respectively [14]. Two different vector backbones were used to test whether this method works with both Zeocin and G418 selection, as was demonstrated in the original paper [15]. Furthermore, multi-copy clones have been shown to linearly increase the titer of intracellular, but not secreted, proteins [5]. Therefore it was interesting to compare the effect of producing GFP as an intracellular protein (pZGFP and pKGFP) or as a secreted protein (pZαGFP and pKαGFP) using the α-mating factor (α-MF) from *Saccharomyces cerevisiae* to direct the protein to the secretory pathway.

All four vectors were transformed into *P. pastoris* and single colonies were selected, expressed in microtiter plates and run on a SDS-PAGE gel to ensure that GFP expression was occurring (data not shown). The Δ*ku70* strain was selected to reduce non-homologous recombination and target the gene of interest to the desired locus (in this case the *AOX1* locus), preventing off target integration of non-functional copies at other sites, which might skew the analysis [18]. Since it has been hypothesized that PTVA utilizes homologous recombination mechanisms, this knockout should have no impact on the PTVA process itself.

**Fig. 1:**
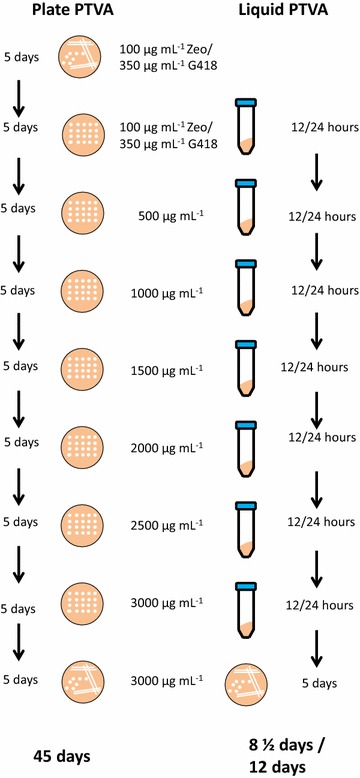
Plate and liquid PTVA methodology. Schematic representation of the method for generating multi-copy clones by traditional plate PTVA (*left*) and liquid PTVA (*right*) changing medium either every 12 or 24 h. Unless otherwise stated concentrations are for both Zeocin and G418 selection

The PTVA procedures are outlined in Fig. 1. Initially colonies that showed the highest level of expression were selected to undergo both traditional PTVA and liquid PTVA where the medium was changed every 12 h (L12). Copy number of the initial clones was determined by qPCR, because clones with different starting copy number can be obtained by transformation, even when plating on low concentrations of antibiotic.

For ease of interpretation each strain is denoted with a number to indicate the initial starting copy number. pZGFP was identified as a single copy clone (pZGFP-1), pZαGFP as a three copy clone (pZαGFP-3), and pKGFP and pKαGFP as two copy clones (pKGFP-2 and pKαGFP-2). For liquid PTVA colonies were inoculated into YPD medium containing the initial antibiotic concentration used for transformation (100 μg mL^−1^ Zeocin and 350 μg mL^−1^ G418). Thereafter cultures were centrifuged every 12 h and the medium replaced with YPD containing the next sequentially higher concentration as indicated in the materials and methods. After the cultures were grown for 12 h in the presence of the highest concentration of antibiotic (3000 μg mL^−1^ for both Zeocin and G418), the cultures were diluted by 10^5^ and plated onto YPD agar plates maintaining selection at 3000 μg mL^−1^.Traditional PTVA was carried out as previously described by Sunga et al., including the initial step which requires streaking to single colony [15]; however to ensure colonies were monoclonal before analysis an additional step was added where the spot from the final plate was streaked to single colonies.

**Fig. 2:**
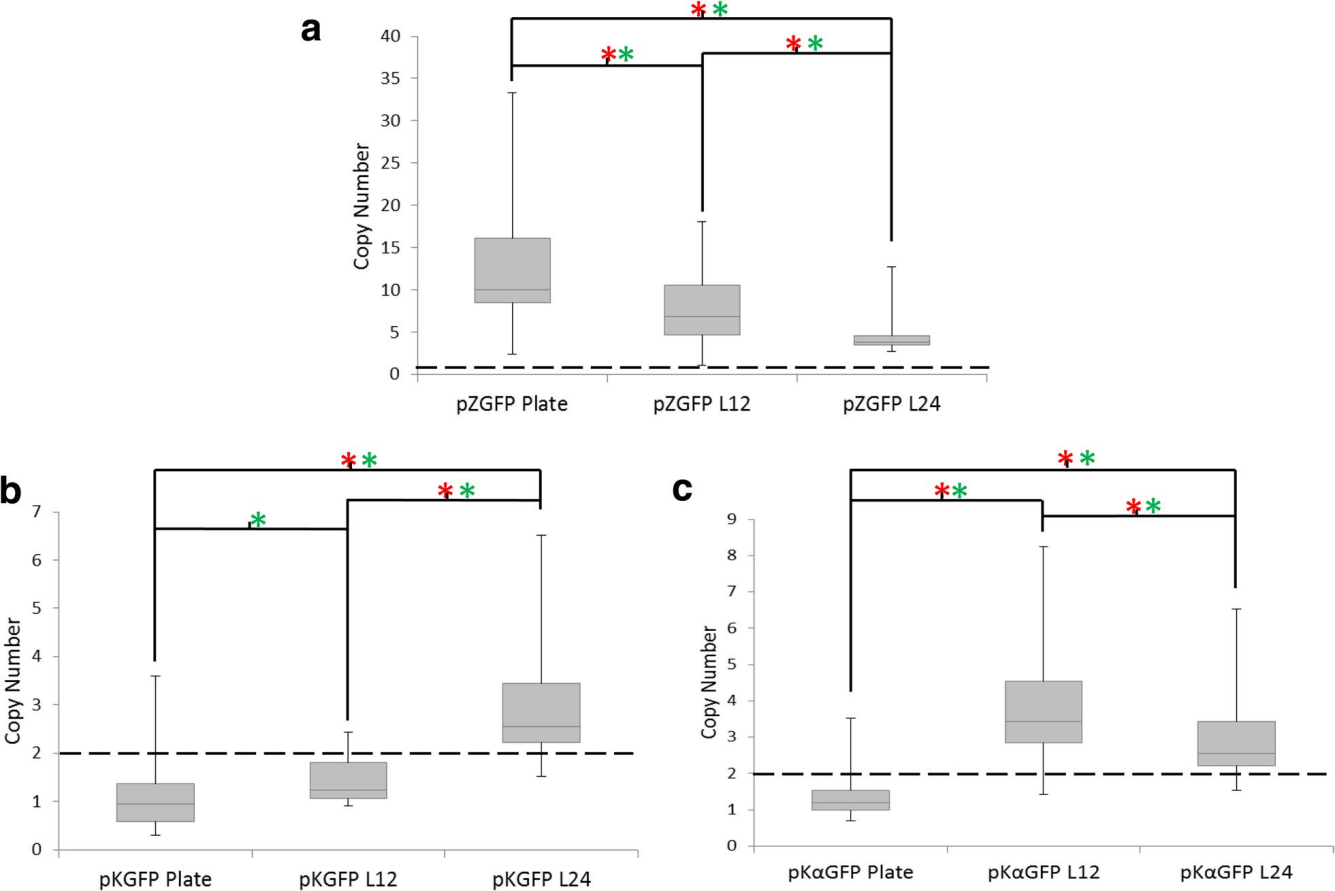
Comparison of copy number after liquid PTVA and plate PTVA. *Box plots* of final copy numbers after PTVA of traditional plate PTVA (Plate), 12 h liquid PTVA (L12) and 24 h liquid PTVA (L24). The *box plots* indicate the first quartile, median and third quartile, with the whiskers indicating the minimum and maximum (n = 20 for each condition). Copy number was determined by qPCR. The *dashed line* indicates the initial copy number of the starting clone. *Red asterisks* indicate significant comparisons according to Kruskal-Wallis one-way analysis of variance (p ≤ 0.05) and *green asterisks* indicate significant comparisons of the median according to Wilcoxon-Mann-Whitney test (p ≤ 0.05). **a** pPICZ-GFP-1 with a starting copy number of one. **b** pKAN-GFP-2 with a starting copy number of two. **c** pKANα-GFP-2 with a starting copy number of two

The first observation was that no colonies were isolated from the pZαGFP-3 culture in the final plating step despite the highest rate of growth during the liquid PTVA experiment, in particular at the lower concentrations of antibiotic. Even though significant cell mass had accumulated over the course of the experiment, growth occurred in the initial stages at low antibiotic concentrations and the cells were non-viable after failing to adapt to the higher concentrations of antibiotics. The liquid PTVA procedure was repeated; however again no colonies were observed (data not shown). From the remaining three conditions a similar number of colonies were obtained (~ 10^7^) from which twenty individual colonies were selected for analysis from both the traditional and L12 PTVA final plates. Each colony was analysed by qPCR to determine copy number (Fig. 2).

For pZGFP-1 (Fig. 2a) the median copy number for strains produced by plate PTVA was significantly higher than that for L12 with an average of 10 copies and 6.8 copies, respectively (p = 0.0123). Furthermore, plate PTVA yielded the strain with the highest individual copy number (37) compared to 18 for L12 and had a significantly larger variation in copy number (p = 0.0119). Despite this, both methods produced a large range of clones with varying copy numbers that should be suitable for conducting a study on the impact of copy number on expression.

The median copy number of pKGFP-2 strains (Fig. 2b) decreased to 0.95 and 1.22 copies for plate and liquid PTVA, respectively, from the initial two copy clone used to start the experiment. Furthermore, none of the strains isolated from L12 had an increased copy number. Plate PTVA did give a small number of strains with a copy number higher than the starting clone (as high as 4). However, statistically there was no significant difference in copy number variance between the two conditions (p = 0.182).

For pKαGFP-2 (Fig. 2c) the median copy number for strains produced by plate PTVA was also lower than the initial starting clone at 1.2; however L12 strains showed a higher median copy number of 3.4. The highest copy clones had 4 and 8 copies of pKαGFP-2 for plate and L12 PTVA, respectively. The increase in both median copy number (p = 8.34E^−8^) and copy number variance (p = 1.69E^−6^) for L12 versus plate PTVA strains was statistically significant.

#### Liquid PTVA with a medium change every 24 h

Due to the lack of isolated colonies from L12 with pZαGFP-3 strains and the fact that liquid PTVA did not consistently result in high copy clones (e.g. pKGFP-1, Fig. 2b), the protocol was modified to change the medium every 24 h (L24) in order to give the cells more time for copy number expansion before forcing adaptation. Furthermore, this is a more manageable experimental set up for a single person as opposed to changing medium every 12 h.

L24 still resulted in no colonies from the pZαGFP-3 experiment when grown on the final selection plate (data not shown). Again, a similar observation was made that growth occurred quickly in the presence of lower concentrations of Zeocin, but the strains failed to adapt to high concentrations.

Colonies from the pZGFP-1, pKGFP-2 and pKαGFP-2 L24 were selected and analysed by qPCR to determine copy number as described previously (Fig. 2). L24 successfully generated multi-copy clones for all three variants. For pZGFP-1 the median copy number from the L24 experiment is the lowest at 3.7, compared to 6.8 and 9.9 for L12 and plate PTVA, respectively (Fig. 2a) and this difference is statistically significant (p = 0.0402, p = 7.5E^−5^, respectively). The maximum copy number observed in a L24 strain was 13 copies, which is also lower than the other two methods. For selection with G418, L24 with pKGFP-2 resulted in both higher median copy number and maximum copy number than both plate and L12 PTVA (Fig. 2b, p = 2.35E^−6^ and p = 3.49E^−6^, respectively). On the other hand, L24 with pKαGFP-2 does not result in clones with higher median copy number than L12 (Fig. 2c).

#### GFP expression from multi-copy clones generated with all three methods

Given the variability in copy number obtained with different experimental protocols, we wanted to determine the GFP titer of strains made by different methods since often the primary goal of undertaking PTVA is to achieve higher expression levels. It has been reported that expression can saturate, meaning that sometimes higher copy number strains are not required [5]. Protein expression was determined using a fluorescence plate reader, analysing either the culture supernatant from strains secreting GFP (pZαGFP and pKαGFP) or the cell pellet for intracellular expression (pZGFP and pKGFP). The latter were normalized using OD_600_ values to account for cellular auto-fluorescence.

**Fig. 3:**
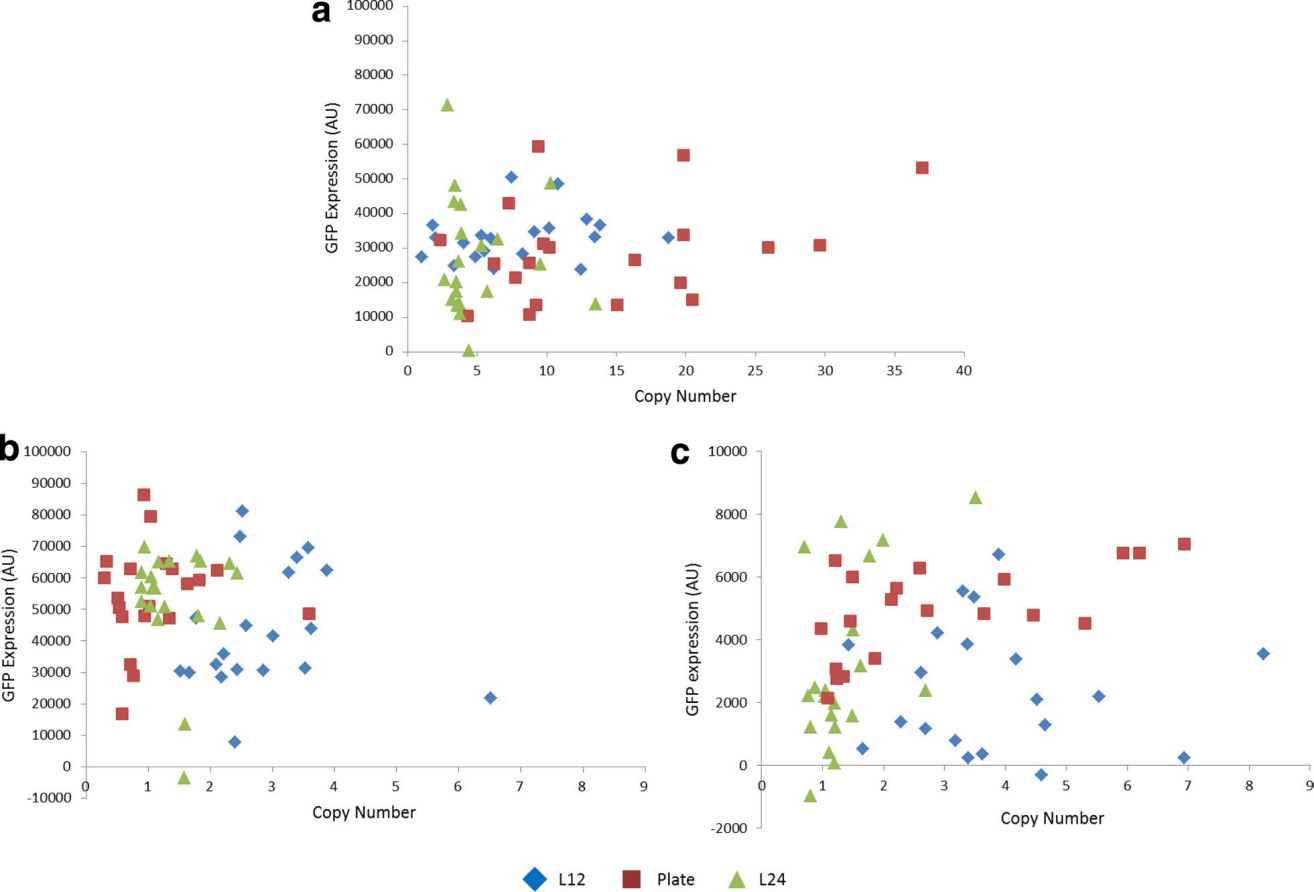
Comparison of GFP expression from strains of different copy numbers created using different PTVA methods. Copy number was measured using qPCR and GFP expression by fluorescence. All three methods of PTVA are presented on each graph, liquid 12 h (L12), liquid 24 h (L24) and traditional plate PTVA. **a** pZGFP-1. **b** pKGFP-2. **c** pKαGFP-2

Copy number does not appear to correlate linearly with titer in any of the strains (Fig. 3). For pKαGFP-2, this observation is in line with previous reports that the secretory pathway can saturate such that after a point, increasing copy number no longer results in further increases in titer for extracellular expression [11, 19, 20]. However, it is more surprising that pKGFP-2 and pZGFP-1, do not show a linear correlation between copy number and expression level, because it has been widely reported that for intracellular expression increased copy number correlates with increased titer [5].

However, even though no general trend of increased titer with increased copy number is observed, highly expressing strains can be obtained with any of the PTVA methods. Moreover, using the Kruskal-Wallis one-way analysis of variance it is apparent there is no significant difference (p > 0.05) in the distribution of GFP expression for the strains generated by either L12 or L24 PTVA compared to their plate PTVA counterparts. Therefore, we suggest that it is possible to use any of the PTVA protocols for the generation of multi-copy clones.

### Starting with a single copy clone increases the success of PTVA

In our initial experiments the starting copy number of the strains varied between one and three copies and this appeared to influence the ultimate copy numbers obtained after PTVA. Specifically, the pZGFP-1 strain, which had an initial copy number of one, gave the clone with the highest copy number after PTVA (37), as well as the highest median copy number of all conditions (Fig. 2a). Moreover, the pZαGFP-3 strain, which had an initial copy number of three failed to yield any clones after PTVA at all. In addition, both the pKGFP-2 and pKαGFP-2, which had an initial copy number of two, showed lower copy numbers after PTVA than the pZGFP-1 (Fig. 2b, c). In order to explore the link between starting and final copy numbers further, we used qPCR to select single copy clones of pZαGFP, pKGFP and pKαGFP and subjected them to traditional plate PTVA and L24.

**Fig. 4:**
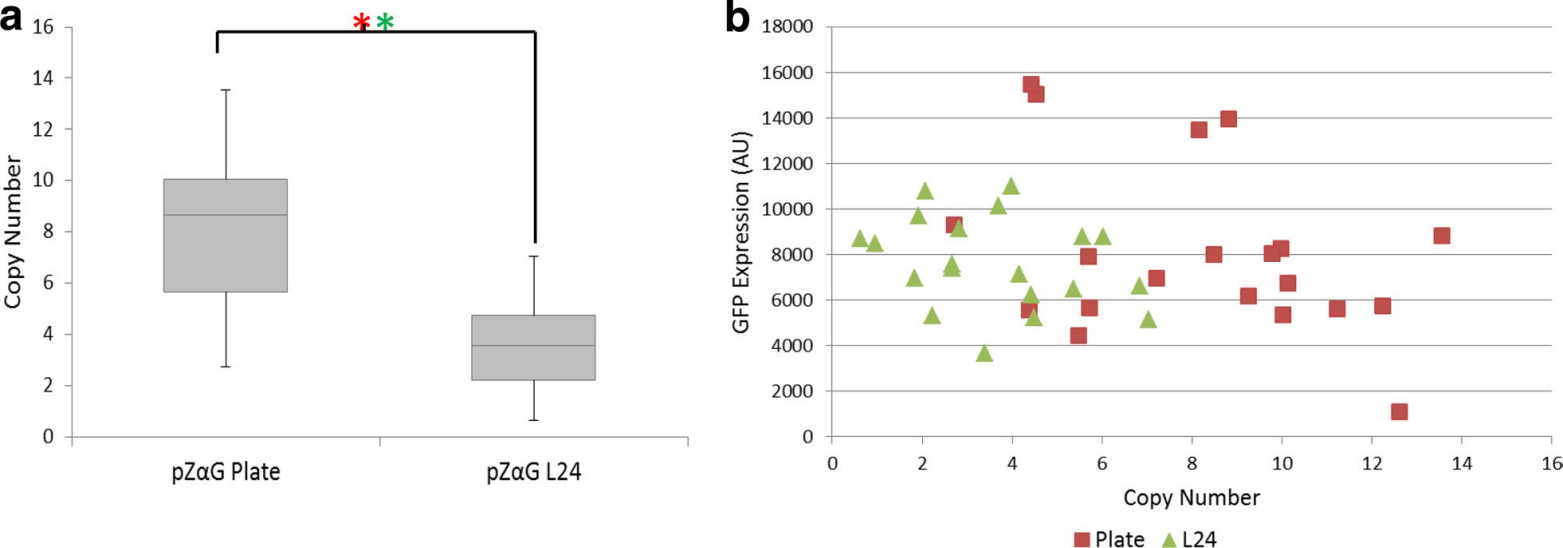
Liquid PTVA was successful for pZαGFP when starting with a single copy clone. **a** *Box plots* of final copy numbers after traditional plate PTVA and liquid PTVA with medium changes every 24 h (L24) of pZαGFP-1. The *box plots* indicate the first quartile, median and third quartile, with the whiskers indicating the minimum and maximum (n = 20 for each condition). *Red asterisks* indicate significant comparisons according to Kruskal-Wallis one-way analysis of variance (p ≤ 0.05) and *green asterisks* indicate significant comparisons of the median according to Wilcoxon-Mann-Whitney test (p ≤ 0.05). Copy number was determined by qPCR. **b** GFP expression against copy number measured by fluorescence for both traditional plate PTVA and L24

First, when starting with a single copy clone, we were able to generate colonies with pZαGFP-1 for both plate PTVA and L24 (Fig. 4), in contrast to the previous experiments. Although median copy number from L24 was not as high as with plate PTVA (3.5 versus 8.6), strains with a range of copy numbers were produced with both methods. The highest copy number achieved with L24 was 7 copies, compared to 13 for plate PTVA.

Median and range of GFP expression levels were not significantly different for strains produced by the two methods (p = 0.881 and p = 0.871 for Wilcoxon-Mann-Whitney and Kruskal-Wallis H tests, respectively). It has previously been reported that saturation can occur at varying copy number depending on the protein being expressed. For instance, with trypsinogen saturation occurs at 2 copies and with human serum albumin at 5–7 copies [7, 10]. There are no reports of saturation levels with this particular variant of GFP (‘superfolder’), but it is apparent from analysing our GFP expression levels (Fig. 3) that a copy number of 7 should be more than sufficient to reach maximum titer (Fig. 4b).

**Fig. 5:**
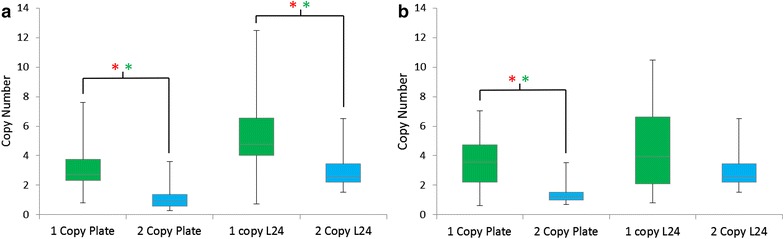
Single copy starting clones result in higher copy number post-PTVA. *Box plots* of final copy numbers after plate PTVA and 24 h liquid PTVA (L24) for two copy clones versus one copy clones. The *box plots* indicate the first quartile, median and third quartile, with the whiskers indicating the minimum and maximum (n = 20 for each condition). Copy number was determined by qPCR. *Blue* indicates copy number of final clones when PTVA was started with a one copy clone strain. *Green* indicates copy number of clones when PTVA was started with a two copy clone strain. *Red asterisks* indicate significant comparisons according to Kruskal-Wallis one-way analysis of variance (p ≤ 0.05) and *green asterisks* indicate significant comparisons of the median according to Wilcoxon-Mann-Whitney test (p ≤ 0.05). **a** pKGFP. **b** pKαGFP

For pKGFP and pKαGFP, it was possible to compare the results of PTVA starting with a two copy strain versus a single copy strain (Fig. 5). Median copy number of strains obtained after PTVA increased when starting with a one copy clone compared to starting with a two copy clone for both PTVA methods. With the pKGFP plasmid, median copy number for strains generated from a one copy clone increased by approximately two copies compared to starting with a two copy clone [2.7 versus 0.9 for plate PTVA and 4.8 versus 2.6 for L24 (Fig. 5a)]. A similar increase was obtained with the pKαGFP plasmid [3.5 versus 1.2 for plate PTVA and 3.9 versus 2.6 for L24 (Fig. 5b)]. Once again there was no correlation between copy number and GFP expression (Additional file 2: Figure S2).

For all three strains tested, it appeared that starting PTVA with a single copy clone resulted in strains with a significantly higher median copy number than when starting with a multi-copy clone. This result was unexpected as the influence of starting copy number has not been previously reported. The observation that single copy clones are better able to expand copy number also supports the theory that our initial pZαGFP-3 clone was unable to adapt to high concentrations of antibiotic because of a lack of selection pressure in early phases of PTVA.

### Liquid PTVA using Zeocin as the selection reagent results in strains with higher growth rates

Some strains generated in this study showed higher titer at lower copy number than other strains with higher copy number. This was also true for the strains expressing intracellular GFP where secretion saturation should not be an issue. Theoretically, due to the nature of liquid PTVA it was possible that the growth rates of the strains could be affected by the need to overcome rapid exposure to higher concentrations of antibiotic. Therefore, we decided to compare the growth rates of some of the strains generated by plate and L24 PTVA to determine if differing growth rates were responsible for the differences in titer. We selected clones from both G418 and Zeocin experiments to determine whether using different antibiotics affected growth rates.

Expression using the *AOX1* promoter often begins with growth in glycerol containing medium for 24 h to accumulate biomass before inducing expression. Hence the cell density after 24 h will give an indication of the culture density prior to induction using methanol. A higher cell density (a larger number of cells) will increase volumetric productivity.

In order to remove any potential burden effects, clones were selected that were of the same or very similar copy number and titer. Growth rates were assessed by taking optical density measurements (OD_600_) on an hourly basis in a glycerol based medium in the absence of antibiotics, as would be the standard protocol for fed batch production. In addition to the initial 9 h time course readings, a final OD_600_ sample was taken after 24 h. Copy number, GFP expression, growth rates and final OD_600_ readings of the selected clones are shown in Table 1.

**Table 1:** Comparison of growth rates and final OD_600_ readings between paired plate and L24 PTVA strains

Strain	Plate PTVA				Liquid PTVA			
	Copy number	Yield (AU)	Growth rate	Final OD_600_	Copy number	Yield (AU)	Growth rate	Final OD_600_
pZGFP	4	69,880	0.2973	59.2	4	66,829	0.4291	65.6
pZGFP	10	211,853	0.31	46	10	187,676	0.4481	66.1
pZαGFP	3	9308	0.3231	46.3	3	9128	0.46	57.3
pZαGFP	7	6951	0.3228	48.2	7	6609.5	0.4283	61.7
pKGFP	2	24,068	0.3447	57.9	2	27,367	0.3474	48.4
pKGFP	6	49,650	0.3443	48.9	7	51,771	0.3426	47.7
pKαGFP	3	4643	0.3335	48.1	3	4789	0.3529	50.9
pKαGFP	7	2858	0.3491	51.1	8	4000	0.3472	47.0

From Table 1 the strains produced by a single method do not appear to differ in growth rate. Therefore, neither additional copies of the integrated gene of interest nor additional copies of the resistance gene impact cellular growth. This implies that there is little to no leaky expression from the *AOX1* promoter before induction with methanol and that multiple copies of the resistance marker, which is constitutively expressed, do not overburden the cells.

The G418 selection strains show very little variation in growth rates or final OD_600_. However, the final OD_600_ of the strains selected via L24 PTVA in Zeocin containing medium are considerably higher than the equivalent plate PTVA strains, as a consequence of a growth rate which is nearly one-third faster. This is likely due to the inherent selection for fast growth in the liquid environment where medium is changed every 24 h, which is absent in plate PTVA where spots are left to grow over a 5 day period. Thus, one advantage of the L24 PTVA method is that it simultaneously selects for strains with faster growth rates and increased copy number. The resulting strains will allow a reduction in total protein production time by minimising the initial batch time frames.

One potential explanation for the increased growth rates of strains selected via L24 PTVA with Zeocin, but not those selected via L24 PTVA with G418 is the mechanism of resistance to the antibiotic. Zeocin induces double stranded DNA breaks, whereas G418 blocks polypeptide synthesis by interfering with the 80S ribosomal subunit [21, 22]. Double stranded breaks in the DNA can result in rapid death. However, faster growing cells may be able to adapt more quickly by increasing the amount of resistance protein because of their overall accelerated rate of protein synthesis [23]. In addition, it is possible that double stranded DNA breaks may also facilitate the duplication of the gene cassette during the PTVA process [24].

On the other hand, impairment of ribosomes may have a slower impact on the cell, particularly those that are not actively dividing [22, 25]. Therefore, slower growing cells are more likely to survive higher concentrations of G418 than faster growing ones. Furthermore it has been reported that in mammalian cells G418 negatively effects growth rate and metabolism [26]. It is also interesting to note that the average copy number of strains selected with G418 is lower than those selected with Zeocin (Fig. 2), which is what might be expected if the selection pressure caused by G418 is weaker. A similar observation has been made in mammalian cells where using a Zeocin resistance cassette resulted in expression of GFP in 100 % of the population, but only 47 % of the population selected with G418 showed expression [27].

## Conclusions

In order to increase the titer of recombinant protein produced in *P. pastoris*, researchers often create strains containing multiple copies of the genes of interest. In addition, for research aimed at increasing the expression capacity of the cell, it is useful to create multi-copy clones with a range of copy numbers in order to systematically evaluate the effects of strain engineering efforts. Therefore a successful, fast and efficient way to do so is required. Various methods have been used historically including plating directly onto selective medium with higher concentrations of antibiotic and in vitro multimerization of the plasmid before transformation [5]. In 2008 Sunga et al. reported a revolutionary method where exposure to a stepwise increase in antibiotic concentration resulted in strains with higher copy number [15]. However, we propose a faster and cheaper alternative to this, which still produces a variety of strains with different copy numbers.

We have shown that it is possible to generate multi-copy clones using a liquid PTVA method with medium changes every 12 or 24 h (L12 or L24, respectively; Figs. 2, 4, 5 and Additional file 3: Table S1). However, L24 resulted in a more robust method that yielded strains with a broader range of copy number for both antibiotics tested (Fig. 2). With L12 multi-copy clones are generated in a total of 8 ½ days. The more effective L24 takes a total of 12 days. In contrast, the original method where each plate is left to grow for 5 days, results in a 45 day period to generate multi-copy clones. Even with the longer L24 protocol, the original method takes over three times as long (Fig. 1).

Additionally, because liquid PTVA can be performed in small volumes, the cost of antibiotics is significantly reduced. Overall less than half the amount of antibiotic is required for the liquid PTVA protocol, irrespective of the timing of the medium changes (Table 2).

**Table 2:** Time and costs of performing traditional plate PTVA and liquid PTVA for both L12 and L24 with Zeocin and G418 selection [28]

	Zeocin			G418		
	Plate PTVA	L12 PTVA	L24 PTVA	Plate PTVA	L12 PTVA	L24 PTVA
Time (days)	45	8 ½	12	45	8 ½	12
Cost (USD)	67.47	32.18	32.18	39.82	18.56	18.56

Costs were determined based on prices on the Thermo Fisher Scientific website

Surprisingly, in the course of our investigation we discovered that the starting copy number of the initial strain influences the final average copy number and the range of copy numbers generated, regardless of the method of PTVA employed. Starting with a single copy clone results in strains with higher median copy numbers post-PTVA than starting with a multi-copy clone, for both liquid and plate PTVA (Fig. 5). To our knowledge, this observation has not previously been reported. Therefore, when beginning PTVA initial colonies should not be selected based on yield, rather a single copy clone should be identified. Perhaps highlighted by the fact that multi-copy clones could only be generated with the pZαGFP vector when starting with a single copy clone (Fig. 4), the underlying cause may be the balance between early growth rate and selection pressure. It is possible that cells with a higher initial copy number can already survive in the early stages of PTVA because the extra copies of the resistance gene are sufficient to survive the initially low antibiotic concentrations, which decreases the amount of vector amplification in early rounds. However, this may later lead to problems if the vector cannot be amplified fast enough to adapt to higher concentrations of antibiotic when challenged. However, as no investigations have been undertaken to determine the precise mechanism of PTVA, it is difficult to explain why a single copy clone is a more advantageous starting point.

Finally, the choice of selection marker was identified as important. Although multi-copy clones were achieved with both selection markers, using Zeocin resulted in higher maximum copy number strains (Figs. 2, 4). In addition, the combination of the new liquid PTVA method and Zeocin resulted in strains with faster growth rates and overall higher OD_600_ (Table 1), which could be beneficial when considering volumetric productivity.

## Methods

### Media and growth conditions

Bacterial strains were cultured in Lennox lysogeny broth (LB) medium (1 % peptone au casein, 0.5 % yeast extract, 0.5 % NaCl) and supplemented with either 100 μg mL^−1^ Zeocin (Life Technologies, Carlsbad, USA) or 50 μg mL^−1^ Kanamycin (Sigma Aldrich, Dorset, UK). Yeast strains were cultured in a rich YPD medium (2 % peptone au casein, 1 % yeast extract, 2 % dextrose). Expression was carried out in buffered minimal glycerol/methanol medium (BMG/BMY; 100 mM potassium phosphate, pH 6.0, 1.34 % yeast nitrogen base, 4 × 10^−5^ % d-Biotin, 1 % glycerol or 0.5 % methanol).

### Strain construction

Bacterial recombinant DNA manipulation was carried out in *Escherichia coli* strain NEB 5-α (New England Biolabs, Hertfordshire, UK). The superfolder-GFP gene of 717 bp was synthesized by GeneArt™ Gene Synthesis (Thermo Fisher Scientific, Paisley, UK; Additional file 4: Figure S3). Initially the GFP was amplified by PCR using Phusion^®^ High-Fidelity DNA polymerase (New England Biolabs) and primers designed to add the correct restriction sites for subsequent cloning (Thermo Fisher Scientific). The PCR fragments were gel extracted using the Zymoclean™ Gel DNA Recovery kit (Zymo Research Corporation, Irvine, USA). The vector pPICZα A (Thermo Fisher Scientific) and the superfolder-GFP were digested with BstBI and Acc65I and ligated to generate the pPICZ-GFP (pZGFP) vector. Alternatively, pPICZα A and superfolder-GFP were digested with PmlI and Acc65I and ligated to generate the pPICZα-GFP (pZαGFP) vector. pKANαB and pKANB were a kind gift from Geoff and Joan Lin-Cereghino (University of the Pacific) and along with GFP were digested with PmlI and *Acc65*I and *Pst*I or *Acc65*I and ligated to form pKANα-GFP (pKαGFP) and pKAN-GFP (pKGFP), respectively. Vectors were ligated with T4 DNA Ligase (New England Biolabs) and transformed into NEB 5-α competent cells (New England Biolabs).

For cloning into *P. pastoris* 5–10 μg of plasmid DNA was linearized with *Pme*I at a single restriction site within the *AOX1* promoter. The vectors were transformed by electroporation according to recommendations in the *Pichia* Expression manual (Thermo Fisher Scientific) into the *P. pastoris* strain *Δku70* (CBS 12694, CBS-KNAW, Fungal Biodiversity Centre, Utretch, The Netherlands) and grown for 3–5 days at 30 °C on either 100 μg mL^−1^ Zeocin (Thermo Fisher Scientific) or 350 μg mL^−1^ G418 (Biochrom Ltd., Cambridge, UK) depending on the vector used.

### Posttransformational vector amplification

Plate PTVA was performed as indicated by Sunga et al. [15] on plates containing 100, 500, 1000, 1500, 2000, 2500 and 3000 μg mL^−1^ Zeocin or 350, 500, 1000, 1500, 2000, 2500 and 3000 μg mL^−1^ G418. An additional final step of streaking out the final spot onto plates containing either 3000 μg mL^−1^ Zeocin or G418 was included to separate mixed cultures within the spot before analysis. For liquid PTVA cells were grown in 5 mL of YPD with the starting antibiotic concentration for either 12 or 24 h at 30 °C shaking at 250 rpm. After the allotted time cells were centrifuged at 4000 rpm at room temperature for 5 min and the supernatant discarded. The medium was then replaced with the next sequential concentration of antibiotic in YPD and left to grow for either 12 or 24 h accordingly. This continued until the highest antibiotic concentration was reached. After the final growth period in 3000 μg mL^−1^ of the relevant antibiotic, cells were diluted by 10^5^ and plated onto a YPD plate containing 3000 μg mL^−1^ antibiotic and left to grow for 3–5 days to obtain single colonies. For both plate PTVA and liquid PTVA a single colony was used for the starting culture.

### Copy number analysis

Genomic DNA was extracted using the DNeasy^®^ Plant Mini Prep Kit (Qiagen, Crawley, UK). Genomic DNA was quantified by Nanodrop™ (Thermo Fisher Scientific) and normalized to 0.5 ng μL^−1^ using distilled H_2_O. Quantitative PCR was run on genomic DNA using SYBR^®^ Green Jumpstart™ *Taq* ReadyMix™ (Sigma Aldrich) in an Eppendorf Mastercycler^®^ ep *realplex* quantitative cycler (Eppendorf UK Ltd, Histon, UK). Copy number was calculated using a standard curve against known concentrations of the plasmid pKAN-GFP. Primers for *GFP* were ATC CGG ATC ACA TGA AAC GC and AAG CTA ATG GTG CGT TCC TG resulting in a 79 bp amplicon. Cycling conditions were 95 °C for 5 min followed by 40 cycles of 95 °C for 30 s, 55 °C for 30 s and 72 °C for 30 s with a melting curve afterwards to ensure a single product was being measured.

### GFP expression

For expression in *P. pastoris*, samples were cultured in 24-deep well plates in 3 mL of BMG (VWR International Ltd, Lutterworth, UK) and sealed with Breathe-Easy^®^ sealing membrane (Sigma Aldrich). Cells were incubated at 30 °C, 216 rpm for 48 h in BMG to allow growth before being centrifuged at 4000 rpm for 5 min. The supernatant was removed and the medium replaced with BMY to induce expression. Cultures were left to express at 20 °C, 216 rpm for 24 h before being harvested. GFP expression was measured on the POLARstar^®^ Omega plate reader (BMG Labtech, Ortenberg, Germany) with excitation at 485/12 nm and emission at 520/30 nm. For intracellular expression samples were normalized according to the OD_600_ of the cultures and corrected for background autofluorescence using a non-transformed control.

### Growth curves

Overnight growth was performed in 50 mL centrifuge tubes with the lids loosely attached to promote aeration. A single colony was used to inoculate 5 mL of YPD and cultured for 16 h at 30 °C, 250 rpm. A 1/1000 dilution from the overnight culture was used to inoculate a 250 mL glass baffled flasks containing 25 mL YPD and cultures were grown at 30 °C, 250 rpm. OD_600_ readings were measured in a Jenway Genova spectrophotometer using YPD as a blank. Readings were taken every hour for the first 9 h, and a final reading at 24 h was also taken. As growth increased cultures were diluted accordingly with YPD prior to the readings.

### Statistical analysis

All analyses were conducted in R [29]. Due to the non-parametric data set the Wilcoxon-Mann-Whitney and Kruskal-Wallis H tests were applied.

### Cost calculation

Costs for Zeocin/G418 usage was based on list prices from the Thermo Fisher website [28]. Initial costs were determined per 1 mL of antibiotic. Each step was calculated in terms of the amount of mL of antibiotic required for a step, 5 mL for liquid PTVA stages and 15 mL for plate PTVA. Total volumes were then multiplied by the cost of 1 mL of antibiotic.

## Additional files

10.1186/s12934-016-0432-8 Schematic representation of the expression vectors pZGFP, pZαGFP, pKGFP and pKαGFP.
10.1186/s12934-016-0432-8 L24 PTVA results in the higher yield of GFP for both pKGFP-1 and pKαGFP-1.
10.1186/s12934-016-0432-8 Summary of PTVA strains.
10.1186/s12934-016-0432-8 DNA Sequence of codon optimized superfolded GFP (sGFP).

## Abbreviations

α-MF: α-mating factor
AOX1: alcohol oxidase 1
L12: 12 h liquid PTVA
L24: 24 h liquid PTVA
OD_600_: optical density at 600 nm
PTVA: posttransformational vector amplification
pKGFP: pKANB + GFP
pKαGFP: pKANBα + GFP
pZGFP: pPICZ + GFP
PZαGFP: pPICZα + GFP
