# Supplementary figures and images for: Liquid PTVA: a faster and cheaper alternative for generating multi-copy clones in Pichia pastoris
Source: Microb Cell Fact. 2016 Feb 5;15:29. doi: 10.1186/s12934-016-0432-8 (PMC4744420; doi:10.1186/s12934-016-0432-8)

A

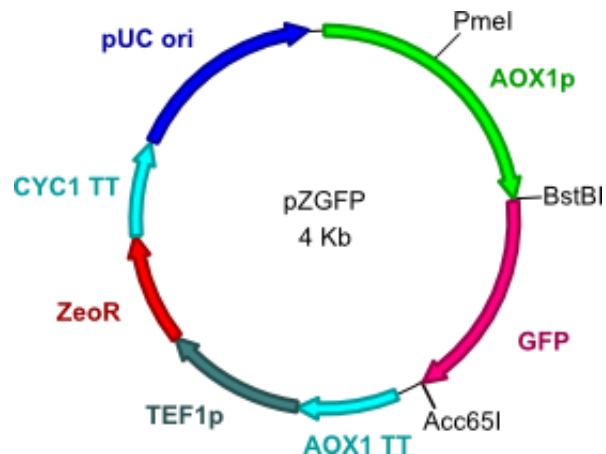

B

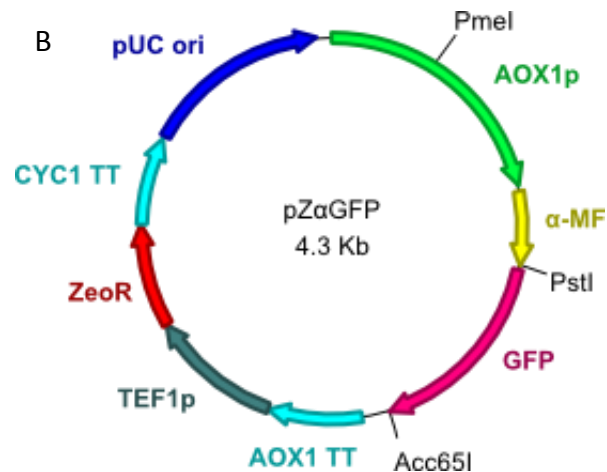

C

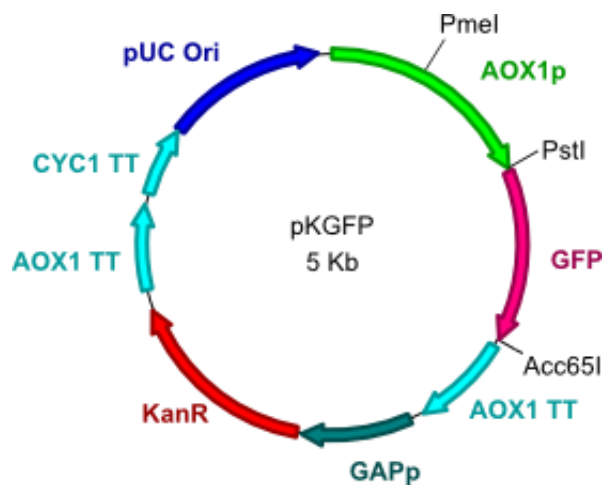

D

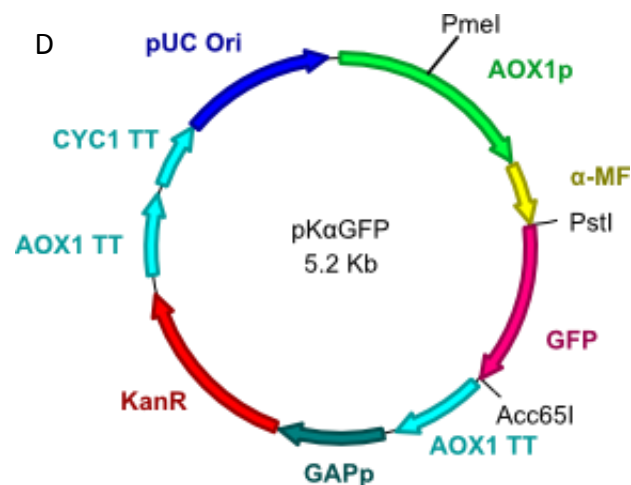

Supplement: Supplementary file 1 — 10.1186/s12934-016-0432-8 Schematic representation of the expression vectors pZGFP, pZαGFP, pKGFP and pKαGFP. [file 12934_2016_432_MOESM1_ESM.pdf]

**A**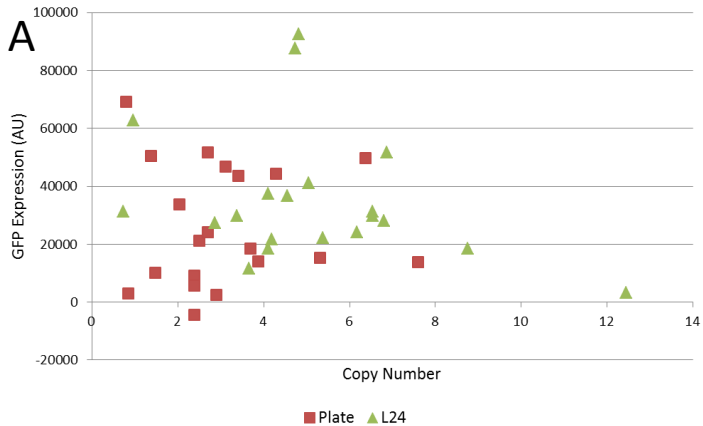**B**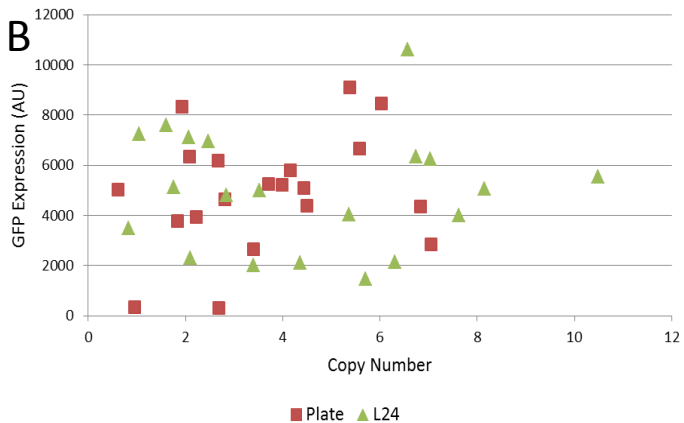

Supplement: Supplementary file 2 — 10.1186/s12934-016-0432-8 L24 PTVA results in the higher yield of GFP for both pKGFP-1 and pKαGFP-1. [file 12934_2016_432_MOESM2_ESM.pdf]
