# Supplementary material for: Liquid PTVA: a faster and cheaper alternative for generating multi-copy clones in Pichia pastoris
Source: Microb Cell Fact. 2016 Feb 5;15:29. doi: 10.1186/s12934-016-0432-8 (PMC4744420; doi:10.1186/s12934-016-0432-8)
Supplement: Supplementary file 4 — 10.1186/s12934-016-0432-8 DNA Sequence of codon optimized superfolded GFP (sGFP). [file 12934_2016_432_MOESM4_ESM.pdf]

```
....|....|....|....|....|....|....|....|....|....|....|....|....|....|....|....|....|....|....|....|
ATGAGCAAAGGCGAAGAACTGTTTACCGGCGTGGTGCCGATTCTGGTGGAACTGGATGGCGATGTGAACGGCCATAAATTTAGCGTGCGCGGCGAAGGCG

       110       120       130       140       150       160       170       180       190       200
....|....|....|....|....|....|....|....|....|....|....|....|....|....|....|....|....|....|....|....|
AAGGCGATGCGACCAACGGCAAACTGACCCTGAAATTTATTTGCACCACCGGCAAACTGCCGGTGCCGTGGCCGACCCTGGTGACCACCCTGACCTATGG

       210       220       230       240       250       260       270       280       290       300
....|....|....|....|....|....|....|....|....|....|....|....|....|....|....|....|....|....|....|....|
CGTGCAGTGCTTTAGCCGCTATCCGGATCACATGAAACGCCATGATTTTTTTAAAAGCGCGATGCCGGAAGGCTATGTGCAGGAACGCACCATTAGCTTT

       310       320       330       340       350       360       370       380       390       400
....|....|....|....|....|....|....|....|....|....|....|....|....|....|....|....|....|....|....|....|
AAAGATGATGGCACCTATAAAACCCGCGCGGAAGTGAAATTTGAAGGCGATACCCTGGTGAACCGCATTGAACTGAAAGGCATTGATTTTAAAGAAGATG

       410       420       430       440       450       460       470       480       490       500
....|....|....|....|....|....|....|....|....|....|....|....|....|....|....|....|....|....|....|....|
GCAACATTCTGGGCCATAAACTGGAATATAACTTTAACAGCCATAACGTGTATATTACCGCGGATAAACAGAAAAACGGCATTAAAGCGAACTTTAAAAT

       510       520       530       540       550       560       570       580       590       600
....|....|....|....|....|....|....|....|....|....|....|....|....|....|....|....|....|....|....|....|
TCGCCATAACGTGGAAGATGGCAGCGTGCAGCTGGCGGATCATTATCAGCAGAACACCCCGATTGGCGATGGCCCGGTGCTGCTGCCGGATAACCATTAT

       610       620       630       640       650       660       670       680       690       700
....|....|....|....|....|....|....|....|....|....|....|....|....|....|....|....|....|....|....|....|
CTGAGCACCCAGAGCGTGCTGAGCAAAGATCCGAACGAAAAACGCGATCACATGGTGCTGCTGGAATTTGTGACCGCGGCGGGCATTACCCATGGCATGG

       710
....|....|....|..
ATGAACTGTATAAATAA
```
